# Army Legislation

**Published:** 1897-01

**Authors:** 


### ARMY LEGISLATION.

THE work of the Army Legislative Committee is daily being enhanced by the help of the profession throughout the country. Under the earnest work of Secretary Morris, of the Empire State Association, every Congressman has been addressed and many seen personally by members of the Association. In Massachusetts, under the direction of member Peters, of the Army Legislative Committee, a strong letter has been sent to members of the Bay State delegation. Presidents Ridge, of the Pennsylvania, and Hart, of the Keystone Veterinary Medical Association, together with State Veterinarian Pearson, have issued a circular letter to all the Keystone Congressmen, and it is to be followed by personal interviews in the several congressional districts by members of the various veterinary organizations in the State. This line of work might be well taken up in every State, and will be strongly felt as a potent influence in favor of this much-to-be-desired measure. Should any member of the profession know of any opposition it will be well to inform the Committee on Army Legislation, so that it may be reached and a knowledge of the causes of such opposition learned.

---

UNDER the head of "Correspondence" there appears in this number of the JOURNAL an important contribution relative to the failure of our national government to properly recognize the value of scientific worth in the Bureau of Animal Industry by a just compensation of those who have filled these all-important places. The Department of Agriculture cannot always remain a school of training for the benefit of our educational institutions without endangering the success of its own work of investigation on behalf of nearly 50 per cent. of our people. This becomes more forcibly true as we learn of projected changes in the *personnel* of the Bureau, which, if true, are freighted with positive danger to the perpetuation of this department. Already there have appeared in the field aspirants for high places, relying on the strength and power of political influence to land them in positions of trust and great responsibility. The experience and fitness of these men for the discharge of the grave duties of these places are wholly secondary to the fact that they can land these plums by the use of the obnoxious pole of so-called party service to greater political bosses.

